# Expression of the arsenite oxidation regulatory operon in *Rhizobium* sp. str. NT‐26 is under the control of two promoters that respond to different environmental cues

**DOI:** 10.1002/mbo3.567

**Published:** 2017-12-17

**Authors:** Paula M. Corsini, Kenneth T. Walker, Joanne M. Santini

**Affiliations:** ^1^ Institute of Structural and Molecular Biology Division of Biosciences University College London London UK

**Keywords:** Agrobacterium, chemotaxis, gene regulation, metabolism

## Abstract

*Rhizobium* sp. str. NT‐26 is a Gram‐negative facultative chemolithoautotrophic arsenite oxidizer that has been used as a model organism to study various aspects of arsenite oxidation including the regulation of arsenite oxidation. The three regulatory genes, *aioX*,* aioS*, and *aioR*, are cotranscribed when NT‐26 was grown in the presence or absence of arsenite. The *aioXSR* operon is upregulated in stationary phase but not by the presence of arsenite in the growth medium. The two transcription start sites upstream of *aioX* were determined which led to the identification of two promoters, the housekeeping promoter RpoD and the growth‐phase‐dependent promoter RpoE2. Promoter–*lacZ* fusions confirmed their constitutive and stationary phase expressions. The involvement of the NT‐26 sigma factor RpoE2 in acting on the NT‐26 RpoE2 promoter was confirmed in vivo in *Escherichia coli*, which lacks a *rpoE2* homolog, using a strain carrying both the promoter–*lacZ* fusion and the NT‐26 *rpoE2* gene. An in silico approach was used to search for other RpoE2 promoters and AioR‐binding motifs and led to the identification of other genes that could be regulated by these proteins including those involved in quorum sensing, chemotaxis, and motility expanding the signaling networks important for the microbial metabolism of arsenite.

## 1 INTRODUCTION

Arsenic (As) is a toxic metalloid and is one of the top 10 chemicals of major public health concern according to the World Health Organization (WHO) (WHO, 2016). Arsenic in the oxidation states arsenite (As^III^) and arsenate (As^V^) are the most common soluble forms found in the environment and both are toxic to organisms (Rosen, 2002). Despite the toxicity of As, a range of phylogenetically diverse prokaryotes are able to survive and thrive in As‐contaminated environments (Stolz, Basu, Santini, & Oremland, 2006).

As^III^ can serve as an electron donor and is oxidized to the less toxic As^V^ with oxygen as the terminal electron acceptor, anaerobically with nitrate (pH > 9) or anoxygenic photosynthesis (Oremland, Stolz, & Saltikov, 2012; Osborne & Santini, 2012). Aerobic arsenite oxidation has been observed in many environments and in a phylogenetically diverse range of prokaryotes (Osborne & Santini, 2012; Stolz et al., 2006). In *Rhizobium* sp. str. NT‐26, As^III^ can be oxidized autotrophically with carbon dioxide as the sole carbon source or heterotrophically with oxygen as the terminal electron acceptor (Santini, Sly, Schnagl, & Macy, 2000).

**Figure 1:**
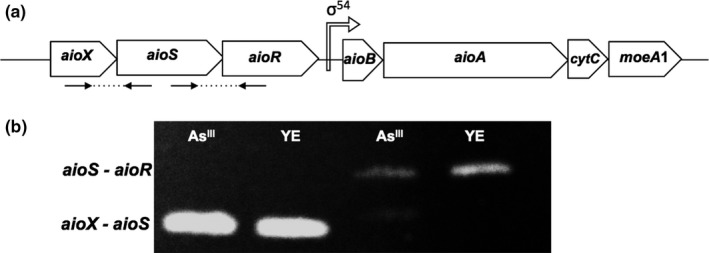
Organization of the NT‐26 *aio* gene cluster. (a) *aioX* encodes periplasmic As^III^‐binding protein; *aioS* encodes sensor histidine kinase; *aioR* encodes transcriptional regulator; *aioB* encodes small subunit of the As^III^ oxidase; *aioA* encodes the large catalytic subunit of the As^III^ oxidase; *cytC* encodes a cytochrome *c*;* moeA*1 encodes a molybdenum cofactor biosynthesis protein. (b) RT‐PCR analysis of the cotranscription of *aioX*–*aioS*, and *aioS–aioR* using *aioXF–aioSR* and *aioSF–aioRR* primers (Table S3) and RNA isolated from NT‐26 grown to late‐log phase with and without As^III^

In NT‐26, As^III^ is oxidized to As^V^ in the periplasm by the As^III^ oxidase (Aio), which is a bioenergetic enzyme that contains a large catalytic subunit (AioA) with a molybdopterin guanine dinucleotide at its active site and a 3Fe–4S cluster, and a small (AioB) Rieske cluster (Santini & vanden Hoven, 2004; Warelow et al., 2013). Homologs of the *aioB* and *aioA* genes have been identified in many phylogenetically diverse prokaryotes including members of the Bacteria and Archaea (van Lis et al., 2013). In many cases, the *aioB* and *aioA* genes are either upstream or downstream of three regulatory genes, *aioX*,* aioS*, and *aioR* (Slyemi, Moinier, Talla, & Bonnefoy, 2013). In NT‐26, the *aioB* and *aioA* genes are in an operon with *cytC* and *moeA1*, downstream of a RpoN promoter (σ^54^), and operon expression is induced by As^III^ (Figure 1a) (Santini et al., 2007). The regulatory genes, *aioX*,* aioS*, and *aioR*, are in a separate operon upstream of *aioB* (Sardiwal, Santini, Osborne, & Djordjevic, 2010), which has been shown to be constitutively expressed (this study). The proposed regulation of *aioB* and *aioA* involves As^III^ sensing by the periplasmic protein AioX and the AioX‐As^III^ complex presumably acts as a ligand for the sensor histidine kinase, AioS, which autophosphorylates and then phosphorylates the transcriptional regulator, AioR, which binds upstream of the RpoN promoter (TGGCACAACGATTGCA) switching on transcription (Andres et al., 2013; Kang, Bothner, Rensing, & McDermott, 2012; Liu et al., 2012; Sardiwal et al., 2010).

AioR appears to play a wider role in regulating gene expression in arsenite oxidizers. Recently, it has also been shown to positively regulate gene expression of the chemotaxis gene *mcp* in *Agrobacterium tumefaciens* GW4 by binding to the *mcp* regulatory region (Shi et al., 2017). The AioR‐binding consensus sequence was also found upstream of the *mcp* gene in NT‐26 and *Herminiimonas arsenicoxydans* ULPAs1 (Shi et al., 2017).

The overall aim of this work was to better understand the physiological roles of AioX, AioS, and AioR in regulating gene expression in NT‐26. To do this, we studied the expression of the *aioX*,* aioS*, and *aioR* genes under different growth conditions using quantitative reverse transcription PCR (qRT‐PCR). We found that the three genes were cotranscribed and that there was an increase in gene expression in stationary phase. Two transcription start sites were identified upstream of *aioX* which resulted in the discovery of two promoters, RpoD (σ^70^) and RpoE2 (σ^24^), that operate under different growth conditions. Promoter functional studies confirmed the differences observed in *aioX*,* aioS*, and *aioR* gene expression under different growth conditions. In silico analyses also implicate the sigma factor RpoE2 in regulating quorum sensing and motility.

## 2 EXPERIMENTAL PROCEDURES

### 2.1 Bacterial culture

A rifampicin‐resistant (Rif^R^) spontaneous mutant of NT‐26 (Santini & vanden Hoven, 2004) was grown in McCartney bottles containing 10 ml minimal salts medium (MSM) containing 0.04% yeast exact (YE) (Oxoid™) with and without 5 mmol/L As^III^ (Santini et al., 2000). Routine transfers were done using a 5% (v/v) inoculum of NT‐26 grown overnight in the respective medium. All cultures were incubated at 28°C under aerobic conditions with shaking at 150 rpm. For the qRT‐PCR and promoter activity experiments, the cells were grown until late‐log (OD_600_ from 0.100 to 0.140) and stationary phases (OD_600_ from 0.170 to 0.24) (Santini et al., 2000). *Escherichia coli* was routinely cultured in lysogeny broth (LB).

### 2.2 Nucleic acid isolation

NT‐26 genomic DNA (gDNA) was isolated using the Wizard^®^ Genomic DNA purification kit (Promega) according to the manufacturer's instructions.

Total RNA was isolated from NT‐26 using the SV Total RNA Isolation System (Promega) following the manufacturer's instructions. To avoid DNA contamination, the DNA‐*free* Kit™ (Ambion) was used according to the manufacturer's instructions and the RNA stored at −80°C. RNA was isolated from five biological replicates for each of the conditions tested by qPCR.

The plasmid pPHU234 (Hübner et al., 1991) and recombinant plasmids were isolated from *E. coli* using the QIAprep Spin Miniprep Kit (Qiagen) according to the manufacturer's instructions.

Nucleic acid concentrations were estimated using a nanodrop spectrophotometer (Thermo Scientific NanoDrop 2000c).

### 2.3 RT‐PCR

The Access RT‐PCR system kit (Promega) was used to confirm the cotranscription of *aioX*,* aioS*, and *aioR* in accordance with the manufacturer's instructions. To confirm that the samples were free of DNA contamination, the RT step was removed and only DNA polymerase was used in the reaction; no PCR products were obtained in these reactions. The primers used in the RT‐PCR are listed in Table S3.

### 2.4 Real time PCR

qPCR were performed using the PikoReal 96 Real‐Time PCR System (Thermo Scientific) using the DyNAmo™ ColorFlash SYBR^®^ Green qPCR Kit (Thermo Scientific). cDNA was synthesized using the RevertAid Premium First Strand cDNA Synthesis kit (Thermo Scientific) and the quantitative PCR (qPCR) First Strand cDNA Synthesis protocol was performed using random primers provided and according to the manufacturer's specifications. The amount of total RNA used to synthesize cDNA was 1 μg and in the qPCR a final concentration of 2 ng/μl of cDNA was used.

The baseline and quantification cycle (Cq) of each reaction was automatically determined using PikoReal Software version 2.1 (Thermo Scientific). The software qBase+ (Hellemans, Mortier, De Paepe, Speleman, & Vandesompele, 2007) was used to analyze the qPCR data to normalize expression levels of the target genes based on the expression of the reference genes *glnA*,* gyrB*, and *gltA*. qBase+ software was also used to calculate the expression level of each gene in the conditions tested, to plot the results using 95% confidence interval, and to perform the analyses of variance (ANOVAs). When comparing two different conditions, the expression of a gene was considered significantly different when *p* < .05 and not significantly different when *p* > .05 based on the ANOVA.

### 2.5 Promoter identification

The SMARTer RACE 5′ kit from Clonetech (Takara Bio Inc., Shiga, Japan) was used to determine the TSS upstream of *aioX* following the manufacturer's specifications. All the reagents, cells, enzymes, and vectors used were provided in the kit aside from the gene‐specific primers designed for *aioX* (Table S3).

DNA samples were sequenced by GATC Biotech (Germany) using the LIGHTrun™ Sanger Technology (GATC Biotech AG). MEGA 6.0 (Tamura, Stecher, Peterson, Filipski, & Kumar, 2013) was used to analyze the sequence chromatograms.

To identify the TSS, sequences obtained from the different cloned fragments (four for each TSS) were aligned to the NT‐26 *aioX* sequence obtained in the MAGE interface (ID: NT26v4_p10026) (Vallenet et al., 2006) using ClustalW (Thompson, Higgins, & Gibson, 1994).

### 2.6 Promoter activity

To test whether the putative promoters upstream of *aioX* were functional, the P_*aioX1*_ and P_*aioX2*_ fragments were PCR amplified using the primers *aioX* p1 and *aioX* p2, forward and reverse, containing *Bam*HI and *Pst*I restriction sites and cloned upstream of a promoterless *lacZ* gene in the plasmid pPHU234 at the *Bam*HI/*Pst*I sites (Hübner et al., 1991). The plasmids were transferred into NT‐26 Rif^R^ by conjugation as described previously (Santini & vanden Hoven, 2004). The promoter assays were performed in *E. coli* strain S17 λ *pir*. To quantify the promoter function, β‐galactosidase activity was measured as described previously (Zhang & Bremer, 1995), and this was done with three biological replicates (Table S4).

### 2.7 In silico search for RpoD‐ and RpoE2‐regulated genes

For the region identified as P_*aioX1*_, the sequences at −35 and −10 were used to build the pattern [TGGACA‐(N)16‐TACAGT]. For the region identified as P_*aioX2*_, the RpoE2‐binding motif described previously for *Sinorhizobium meliloti* (Sauviac et al., 2007) was identified by eye and used to construct the pattern [GGAAC‐(N)18‐TT‐(N)8‐G]. The two motifs were used as input for the “Find Individual Motif Occurrences” tool (FIMO) (Grant, Bailey, & Noble, 2011), which matched the motifs against a library of upstream regions (up to 400 nucleotides in length from the starting ATG) for every gene in NT‐26. The resulting alignments were used to generate a summary motif with MEME/MAST (Bailey & Elkan, 1994).

## 3 RESULTS

### 3.1 Cotranscription of the *aioX*,* aioS*, and *aioR* genes in NT‐26

To determine whether the *aioX*,* aioS*, and *aioR* genes were cotranscribed in NT‐26, and therefore, part of the same operon, RT‐PCR was performed using the RNA isolated from NT‐26 grown heterotrophically either in the presence or absence of As^III^ (Figure 1b). Two sets of primers were used, one to amplify the 3′ end of *aioX* and the 5′ end of *aioS* and, and the second to amplify the 3′ end of *aioS* and the 5′ end of *aioR* (Figure 1a, see arrows). The results demonstrate that *aioX*,* aioS*, and *aioR* are cotranscribed under both conditions.

### 3.2 The effect of As^III^ and growth phase on *aioX*,* aioS*, or *aioR* expression

**Figure 2:**
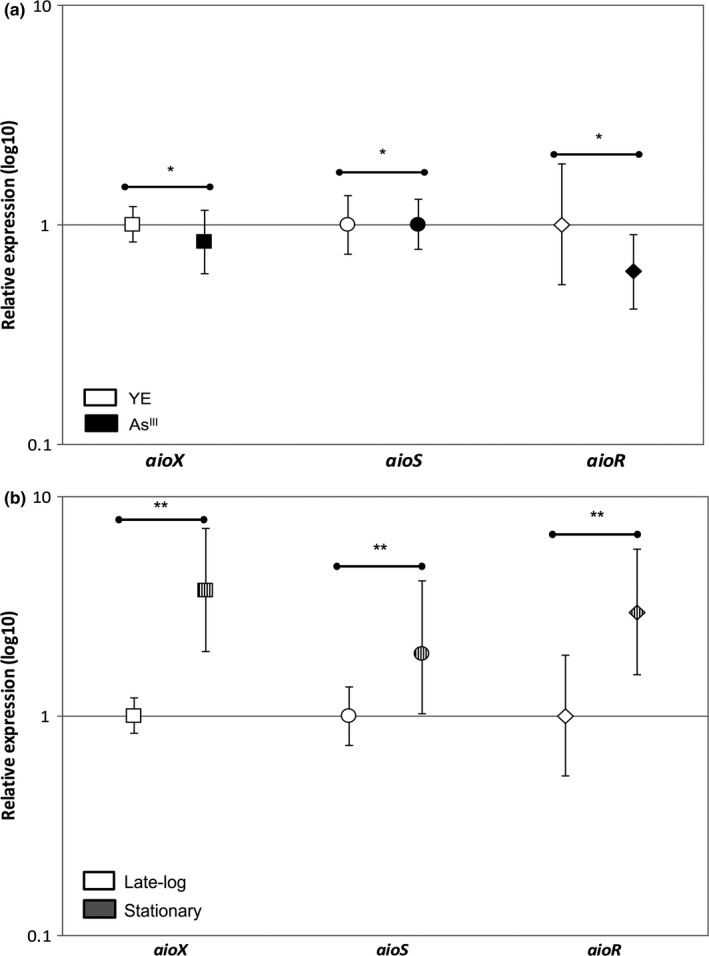
Relative expression analysis using qPCR to compare the expression ratios. (a) Samples from NT‐26 grown heterotrophically (with 0.04% yeast extract, YE) with and without As^III^ in the growth medium. The error bars show the 95% upper and lower confidence intervals and *p > .05. (b) Samples from NT‐26 grown heterotrophically without As^III^ grown to late‐log or stationary phase. The error bars show the 95% upper and lower confidence intervals. **p < .05

As determined by qRT‐PCR, As^III^ had no effect on the expression of *aioX*,* aioS*, and *aioR* (Figure 2a). The qPCR was normalized using the reference genes that encode glutamine synthetase (*glnA*), citrate synthetase (*gltA*), and DNA gyrase subunit B (*gyrB*). These reference genes were selected based on their expression stability in NT‐26 when grown heterotrophically in the presence and absence of As^III^ in late‐log and stationary phases.

In NT‐26, the *aioX*,* aioS*, and *aioR* genes were upregulated in stationary phase of growth when compared to late‐log phase. The increase in expression was statistically significant (with *p* < .05) with increases of 3.7‐fold for *aioX*, twofold for *aioS*, and threefold for *aioR* (Figure 2b and Table S1).

### 3.3 Identification of two transcription start sites and the associated promoters upstream of *aioX*

**Figure 3:**
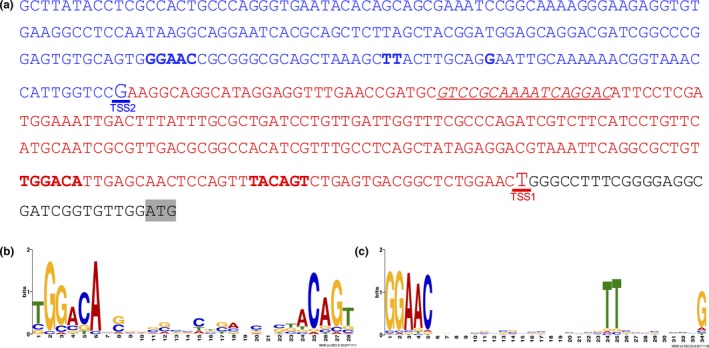
Identified promoters upstream of *aioX*. (a) The two identified transcription start sites (TSSs) upstream of *aioX* are underlined, the TSS1 is underlined in red and the TSS2 is underlined in blue. The six nucleotides flanking the −10 and −35 regions upstream of TSS1 correspond to the RpoD conserved region are in red and in bold. The predicted AioR‐binding site is shown italicized and underlined. The conserved regions for the RpoE2 promoter are shown in blue and in bold. (b) The RpoD promoter conserved site motif built with MEME/MAST using conserved nucleotides in NT‐26. (c) The RpoE2 promoter conserved site motif built with MEME/MAST using conserved nucleotides in NT‐26

To determine whether the *aioXSR* operon is under the control of one or more promoters, the transcription start site(s) (TSS) upstream of *aioX* was determined using 5′ RACE when NT‐26 was grown heterotrophically until late‐log or stationary phase. Two different TSS were identified: (1) the proximal one named TSS1 and (2) the distal one named TSS2 (Figure 3a). The untranslated region (UTR) was identified and the TSS corresponds to the first 5′ nucleotide of the UTR. These were named UTR1 and TSS1 for the proximal TSS (highlighted in red), and UTR2 and TSS2 for the distal one (highlighted in blue). The presence of two different TSS suggests that this operon is regulated by two different promoters.

To identify the promoters, the two regions upstream of both TSSs were subjected to visual inspection and in silico analysis. For the constitutively expressed region, six nucleotides spanning the −10 and −35 regions upstream of TSS1 were selected (Figure 3a) and used to construct the nucleotide pattern [TGGACA‐16‐TACAGT] (Figure 3b) which has been previously shown to be a consensus sequence for a RpoD promoter (Harley & Reynolds, 1987). This consensus sequence was found upstream of 176 different genes in NT‐26 (Table S2) some of which are involved in nitrogen fixation, primary metabolism, and other cellular functions associated with RpoD promoters (Ramírez‐Romero, Masulis, Cevallos, González, & Dávila, 2006). Upstream of the putative RpoD promoter we also identified the predicted binding site for AioR (underlined in Figure 3a) (Andres et al., 2013).

Visual inspection of the region upstream of TSS2 revealed a nucleotide pattern, GGAACN16‐17cgTT, similar to the RpoE2‐binding site in *Rhizobium meliloti* (Figure 3a) (Sauviac, Philippe, Phok, & Bruand, 2007). Since the RpoE2‐controlled promoters are upregulated during cellular stress (Bastiat, Sauviac, & Bruand, 2010), the presence of an RpoE2 promoter‐binding site might imply that the *aioXSR* operon is upregulated during stationary phase as a general stress response.

The putative RpoE2 promoter‐binding site was used to construct the nucleotide pattern [GGAAC‐(N)18‐TT‐(N)8‐G] (Figure 3c), and used to search the NT‐26 genome for other genes possibly regulated by the RpoE2 sigma factor. The RpoE2 promoter‐binding motif was found upstream of 469 genes in NT‐26 (Table S2); such a high number of hits suggest the motif was too generic. Nevertheless, the NT‐26 RpoE2 promoter motif was found upstream of the *rpoE2* gene, which is also the case in *R. meliloti* (Sauviac et al., 2007). In addition, the RpoE2‐binding motif was found upstream of putative genes involved in chemotaxis and motility, *qseB* and *fliG*, respectively.

### 3.4 Functional analysis of the RpoD and RpoE2 promoters in NT‐26 using reporter gene fusion

**Figure 4:**
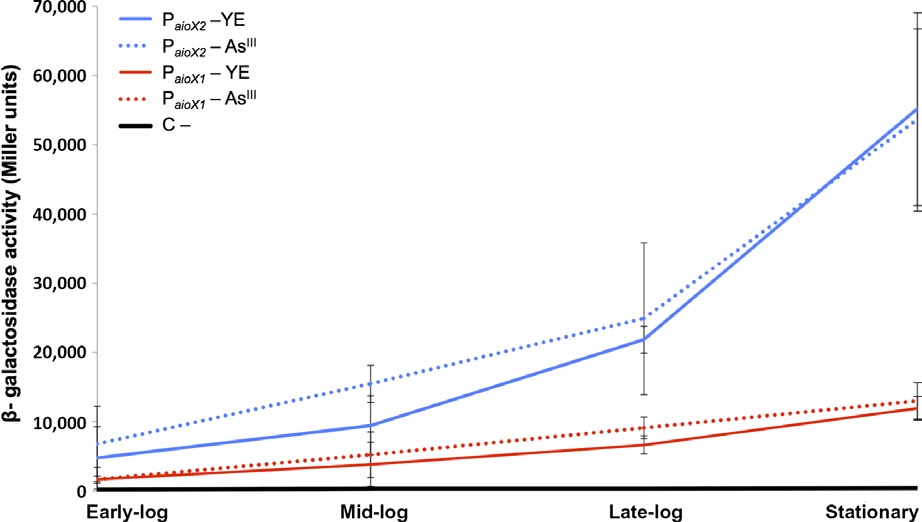
β‐Galactosidase activity determined at different growth stages for the *lacZ*–promoter in NT‐26 grown heterotrophically (with 0.04% yeast extract, YE) with and without As^III^. The negative control samples (i.e., the promoterless pPHU234 plasmid) are shown in black. The β‐galactosidase activity for NT‐26 containing the RpoE2 promoter (P_*aioX1*_) in trans is shown in blue and NT‐26 containing the RpoD promoter (P_*aioX2*_) in trans is shown in red. The data plotted correspond to the average of three independent experiments

To verify the function of the RpoD and RpoE2 promoters in regulating the *aioXSR* operon in NT‐26, the region upstream of the TSS1 and TSS2, designated P_*aioX1*_ and P_*aioX2*_, were cloned upstream of a promoterless *lacZ* gene in the plasmid pPHU234 (Hübner et al., 1991). The plasmid was transferred into NT‐26 by conjugation and β**‐**galactosidase activity monitored over the course of growth, with samples taken at early‐log (OD_600_ 0.030–0.058), mid‐log (OD_600_ 0.07–0.098), late‐log (OD_600_ 0.115–0.140), and stationary (OD > 0.200) phases (Figure 4).

Results of the promoter function assays are reported in Figure 4, with β‐galactosidase activity in units plotted against growth phase to observe if there is a link between the growth phase and the activity of the putative promoters. NT‐26 harboring the vector pPHU234 alone served as the negative control and as expected there was no detectable β‐galactosidase activity (Figure 4). The activity of the P_*aioX2*_–*lacZ* gene fusion displayed increased β‐galactosidase activity over time with the highest activity detected in stationary phase. As expected the P_*aioX1*_ *–lacZ* fusion was constitutively expressed with no significant increase in activity over time.

### 3.5 The NT‐26 RpoE2 is required for activity of the P_*aiox2*_ promoter in *Escherichia coli*

**Figure 5:**
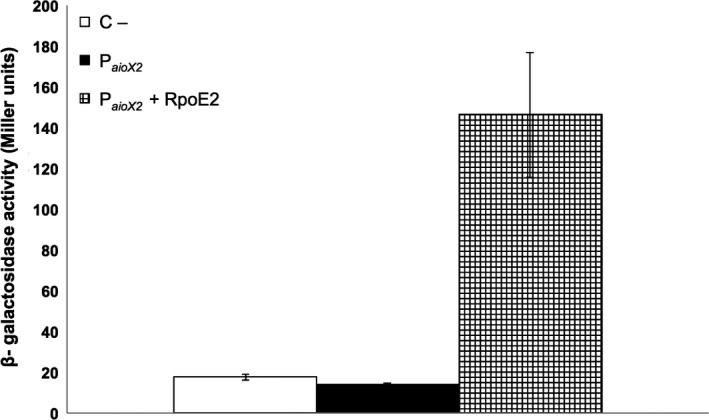
β‐Galactosidase activity determined for the *lacZ*–promoter fusions also harboring the NT‐26 *rpoE2* gene in *Escherichia coli*. C, negative control corresponds to the activity of the promoterless plasmid pPHU234; P*aioX1*, negative control of the RpoE2 promoter alone; P_*aioX1*_ + RpoE2, RpoE2 promoter and also a plasmid harboring the NT‐26 *rpoE2* gene. The β‐galactosidase activity plotted is the average of three independent experiments

To confirm the involvement of the NT‐26 sigma factor RpoE2 in regulating the *aioXSR* operon, *E. coli*, which does not naturally contain a *rpoE2* homolog, was used as a host for in vivo experiments. *E. coli* containing P_*aioX2*_ alone showed no detectable β‐galactosidase activity (Figure 5), however, when a plasmid containing the NT‐26 *rpoE2* gene was also provided in trans β‐galactosidase activity was detected (Figure 5).

## 4 DISCUSSION

It has been previously shown that the *aioX*,* aioS*, and *aioR* genes are essential for As^III^ oxidation and expression of the arsenite oxidase genes in NT‐26 (Andres et al., 2013; Sardiwal et al., 2010). The AioX, AioS, and AioR proteins are thought to be involved in a three‐component system involved in the regulation of the *aioB* and *aioA* genes in the presence of As^III^ in the growth medium (Andres et al., 2013; Sardiwal et al., 2010). In this study, we have shown that the *aioX*,* aioS*, and *aioR* genes are cotranscribed and that there is no effect on expression of these genes when NT‐26 was grown in the presence of As^III^. Similar results have also been reported for *Thiomonas arsenitoxydans* 3As, where the presence or absence of As^III^ had no effect on the expression of *aioX*,* aioS*, or *aioR* (Slyemi et al., 2013). However, in *A. tumefaciens* 5A, the presence of As^III^ induces the expression of *aioX* (Liu et al., 2012), and in *H. arsenicoxydans* ULPAS‐1, *aioX*, aioS, and *aioR* are all upregulated after 8 hr exposure to As^III^ (Cleiss‐Arnold et al., 2010).

In NT‐26, a growth‐phase‐dependent effect on expression was observed where the *aioX*,* aioS*, and *aioR* genes were upregulated in stationary phase (Figure 2). These results can be explained by the detection of two different TSSs and the identification of two promoters, RpoD and RpoE2. The TSS upstream of *aioX* in *T. arsenitoxydans* 3As was also determined and a RpoD consensus sequence identified (Moinier et al., 2014). Given the constitutive expression of the *aioXSR* operon, the identification of a RpoD promoter was expected. Perhaps surprisingly, the *aioXSR* operon was upregulated in stationary phase resulting in the identification of a RpoE2 promoter. In *R. meliloti*, the RpoE2 sigma factor is involved in the general stress and starvation response (Sauviac et al., 2007), and this may also be the case in NT‐26.

The involvement of RpoE2 in regulating the *aioXSR* operon in stationary phase in NT‐26 helps us elucidate the link between the regulation of As^III^ oxidation, motility, and quorum sensing that has been previously suggested for NT‐26 (Andres et al., 2013), *Agrobacterium* GW4 (Shi et al., 2017), *A. tumefaciens* 5A (Kashyap, Botero, Franck, Hassett, & McDermott, 2006), and *H. arsenicoxydans* ULPAs1 (Muller et al., 2007). The RpoE2‐binding consensus sequence was also found upstream of the genes *rpoe2*,* kat*, and *qseB* (refer to Table S2), the latter two of which encode putative proteins involved in the response to oxidative stress and flagella regulation, respectively. In NT‐26, the *kat* gene was also found to be upregulated by As^III^ (Andres et al., 2013), and in *Sinorhizobium meliloti*, it is known to be regulated by RpoE2 (Sauviac et al., 2007). In *E. coli*, the *qseB* gene encodes a putative regulatory protein involved in quorum sensing and flagella gene expression (Sperandio, Torres, & Kaper, 2002), and was also shown in NT‐26 to be upregulated by As^III^ (Andres et al., 2013). NT‐26 was also shown to be more motile when grown in the presence of As^III^, reinforcing the link presented here between the regulation of *qseB* and the *aioXSR* operon regulation by RpoE2 (Andres et al., 2013). We hypothesize that in stationary phase when the As^III^ concentration is reduced (Santini et al., 2000), a greater abundance of AioX, AioS, and AioR would allow NT‐26 to sense and respond to lower concentrations of As^III^. This together with the As^III^‐induced regulation of chemotaxis would facilitate its motility toward As^III^.

In NT‐26, the consensus sequences for the predicted AioR‐binding site was found upstream of the putative chemoreceptor‐encoding gene, *mcp* (Shi et al., 2017). Mcp is a chemoreceptor described in *A. tumefaciens* GW4 to bind As^III^ and it has been shown to be important for chemotaxis toward As^III^ in this organism (Shi et al., 2017). In NT‐26, it seems that Mcp may also be involved in chemotaxis toward As^III^ and that *mcp* expression is under the control of AioR, further strengthening the link between As^III^ sensing, As^III^ oxidation, and As^III^‐induced chemotaxis. The chemotaxis genes, involved in regulating the activity and direction of the flagella (Wadhams & Armitage, 2004), *cheY*,* cheR*,* cheW*, and *cheD* also contain a putative RpoE2‐binding site in their promoter regions (Table S2) (no RpoD promoters were identified).

The presence of As^III^ in the growth medium has no effect on the expression of the *aioXSR* operon, however, the results presented herein introduces possible links between the regulation of As^III^ oxidation, motility, and quorum sensing, and opens up the possibility that the regulatory proteins AioX, AioS, and AioR may have other roles other than regulating As^III^ oxidation in NT‐26. The results presented here also suggest the involvement of the sigma factor RpoE2 in the regulation of As^III^ oxidation and the link to chemotaxis and confirms the involvement of RpoD in regulating expression of the *aioXSR* operon as first suggested for *T. arsenitoxydans* 3As (Moinier et al., 2014).

## CONFLICT OF INTEREST

The authors declare no conflict of interest.
